# Safety Assessment of Water-Extract Sericin from Silkworm (*Bombyx mori*) Cocoons Using Different Model Approaches

**DOI:** 10.1155/2020/9689386

**Published:** 2020-11-05

**Authors:** Huiyan Qin, Jiehong Zhang, Hui Yang, Siyu Yao, Li He, Huili Liang, Yanwu Wang, Huafeng Chen, Peng Zhao, Guangqiu Qin

**Affiliations:** ^1^Institute of Toxicology, Guangxi Center for Disease Prevention and Control, Nanning, China; ^2^Department of Preventive Medicine, Guangxi University of Chinese Medicine, Nanning, China

## Abstract

Sericin is a natural protein component of silks of silkworm and has potential utility in multiple areas such as pharmacological, cosmetics, and biotechnological industries. However, the understanding of its toxicological safety is still limited. This study evaluated the safety of water-extract sericin from silkworm (*Bombyx mori*) cocoons using different model approaches, including three genotoxicity studies (the bacterial reverse mutation test, the mammalian erythrocyte micronucleus test, and the mouse spermatogonia chromosomal aberration test) and a 90-day subchronic toxicity study in Sprague-Dawley (SD) rats. The results of this study showed that water-extract sericin was nonmutagenic and nongenotoxic both *in vitro* and *in vivo*. Sericin did not induce significant changes in the body and organ weight, food intake, blood hematology and serum biochemistry, urine index, and histopathology in rats. The NOAEL of sericin was determined to be 1 g/kg/day for male and female rats. These results indicated that water-extract sericin was of low toxicity in the experimental conditions of the current study and had the potential for application in food-related products.

## 1. Introduction

Sericin is a natural mixture of proteins synthesized by silkworm (*Bombyx mori*) larvae. Natural silk consists of two main proteins, sericin and fibroin, with fibroin being the structural center and sericin being the surface coating surrounding it. Sericin is essential in the formation of a cocoon. However, since sericin affects silk fabric dyeing and feel of silk floss, it is removed during the degumming or refining of raw silk. In the silk of silkworms, sericin accounts for about 20-30% of the weight and fibroin accounts for the rest [1]. The molecular weight of sericin was between 10 and 400 kDa [2, 3]. A total of 18 kinds of amino acids have been found in silkworm sericin, among which polar amino acids account for about 78% (mainly serine and aspartic acid) and nonpolar amino acids account for 22% [4].

Silkworm cocoon and its water extract have been used as a component of Chinese medicine to treat diabetes-related diseases for centuries. In East Asia, silkworm cocoon and its boiled water (mainly sericin) are used as an important component in diet therapy regimens to treat hyperglycemia and hyperlipidemia. The results of modern biomedical studies showed that sericin in silkworm cocoons has multiple biological activities. For example, sericin was observed to activate collagen synthesis in skin tissue and play the role of antiwrinkle and antiaging through its collagen-promoting activity [5–7]. Sericin could stimulate cell proliferation in serum-free culture dishes [4, 8]. Other studies found that sericin reduced the serum lipid content and improved the glucose tolerance in rats fed with high-fat diet [9, 10] and had protective effects on alcohol-induced liver injury in mice [11]. These results suggest that sericin has a wide application potential and it has been applied in food-related products, cosmetics, and medical supplies [12].

Although the biological effects of sericin in silkworm cocoon have been widely recognized in public and confirmed by modern biomedical research, the understanding of its toxicological safety is still limited. Previous studies have reported adverse effects of sericin such as immunologic stimulation and cytotoxicity [13, 14]. For example, it was reported that sericin extracted by the urea treatment was severely harmful to cells at concentrations > 100 *μ*g/mL [5]. The objective of the present study was to evaluate the genotoxicity and subchronic toxicity of water-extract sericin from silkworm cocoon using different model approaches, so as to provide necessary information for safety assessment sericin in food-related products.

## 2. Materials and Methods

### 2.1. Sericin Extract and Its Characterization

Fresh *Bombyx mori* cocoons were provided by the Guangxi Institute for Product Quality Inspection (Nanning, China). A traditional high-temperature degumming technique was used to prepare heat-degraded silk sericin solution. Briefly, cocoons were cut into small pieces and immersed in deionized water (1 : 30, w : v) and degummed at 100°C for 3 h, without chemical additives. The silk sericin solution was filtered with an 18-mesh nonwoven filter to remove the silk fibroin, and the sericin solution was further boiled to concentrate the sericin. The concentrated solution was centrifuged (8000 rpm) for 10 min. The supernatant was collected and freeze-dried to obtain sericin extract. Sericin extract was dissolved in deionized water before used for the study.

The amino acid compositions of the water-extract sericin were examined in accordance with the standardized guidelines [15]. Briefly, the samples were hydrolyzed with 6 mol/L HCl (1 : 3, w : v) and 0.2 mL phenol at 110°C for 22 h under vacuum. Amino acids were analyzed with a Hitachi L-8500A amino acid analyzer (Tokyo, Japan).

### 2.2. Experimental Animals and Husbandry

Three-week-old specific pathogen-free Sprague-Dawley (SD) rats and adult Kunming mice (25-35 g) were provided by the Animal Experimental Center at Guangdong Academy of Medical Science (Guangzhou, China). The animals were kept in the laboratory animal facility with a controlled temperature at 23 ± 1°C, relative humidity at 60 ± 5%, and a 12 h light/dark cycle. The animals were offered conventional diets and sterilized tap water *ad libitum*. The animals were acclimated for one week before used for the experiments. All animal test protocols were approved by the Animal Experimentation Ethics Committee at Guangxi Center for Disease Prevention and Control (Nanning, China). The data of approval was 2017-05-08, and the approval number was 20170009.

### 2.3. Genotoxicity Studies

To evaluate the potential genotoxicity of water-extract sericin, the bacterial reverse mutation test, the mammalian erythrocyte micronucleus test, and the mouse spermatogonia chromosomal aberration test were conducted. The doses used in these studies were selected based on the maximum tolerated dose (MTD) for oral intake of water-extract sericin in the preexperiment and following the recommendation of standardized guidelines set by the Ministry of Health of China. These guidelines were developed based on the internationally recognized guidelines including the OECD Guidelines for the testing of chemicals and USFDA Redbook 2000 Toxicological Principles for the Safety Assessment of Food Ingredients [16, 17].

The bacterial reverse mutation test was conducted to examine the ability of water-extract sericin to induce the reverse mutation at five strains of *Salmonella typhimurium* (TA97a, TA98, TA100, TA102, and TA1535), following the standardized protocol described previously [16, 18]. A maximum dose of 5000 *μ*g/plate for potentially low bacterial toxicity substances was used as the highest dose, followed by 1581, 500, 158, and 50 *μ*g/plate, using a common interval ratio of √10. Rat liver S9 mix (MoltoxVR Molecular Toxicology, Inc., USA) was used as the exogenous metabolic activation, and five standard mutagens were used as the positive control, including 10 *μ*g/plate 2-aminofluorene, 6 *μ*g/plate daunorubicin, 50 *μ*g/plate dexon, 1.5 *μ*g/plate sodium azide, and 50 *μ*g/plate 1, 8-dihydroxyanthraquinone. The test solutions were autoclaved (0.103 MPa, 20 min) before used for the test. Using the plate mixing method, 0.1 mL of the test *S. typhimurium* solution, 0.1 mL of water-extract sericin, and 0.5 mL of S9 mixed solution (when metabolic activation was needed) were successively mixed with the top layer of culture medium and then poured into the hardened bottom culture medium. For solvent control, sterilized pure water and dimethyl sulphoxide (DMSO) were used instead of water-extract sericin, and other conditions were the same as those of the treatment groups. Three parallel dishes were made for each dose group. The bacteria were cultured at 37°C for 48 hours, and the number of colonies in each dish was counted. The bacterial reverse mutation test was repeated twice under the same experimental conditions.

The *in vivo* mammalian erythrocyte micronucleus test was conducted following the standardized method described previously [19, 20]. Briefly, 25-30 g Kunming mice were randomly assigned to 5 groups with 5 males and 5 females in each group. The animals in the 3 treatment groups were treated orally with 6660, 3330, and 1665 mg/kg sericin extract twice with a 24 h interval, while the animals in the negative and positive controls were treated with distilled water and 40 mg/kg cyclophosphamide, respectively. After 6 h following the second gavage, the peripheral blood of the animals was collected from the caudal vein and fixed on slides. For each animal, 2000 polychromatic erythrocyte PCEs were counted, and the micronucleus rate was expressed by the percentage of PCEs with micronuclei. At the same time, the polychromatic erythrocytes (PCE) observed in 1000 red blood cells (RBCs) were counted, and the PCE/RBC ratio was calculated.

The mouse spermatogonia chromosomal aberration test was conducted following the standardized protocol [21]. Briefly, 25-30 g male Kunming mice were randomly divided into 5 groups with 10 animals in the high dose group (6660 mg/kg) and 5 animals in each of the rest group. The animals in the 3 treatment groups were treated by oral gavage with 6660, 3330, and 1665 mg/kg sericin extract once. The animals in the negative and positive groups received distilled water and 40 mg/kg cyclophosphamide, respectively. Twenty-four hours after the exposure, 5 animals in each group were sacrificed, while 48 h after the exposure, 5 animals in the high dose group were sacrificed for spermatogonia chromosomal aberration examination. Three hours before sacrificed, the animals were intraperitoneally injected with 4 mg/kg colchicine at a dosing volume of 10 mL/kg. After sacrificed by cervical dislocation, the testes were collected and seminiferous tubules were soaked in 1% trisodium citrate, fixed in 75% methanol (containing 25% glacial acetic acid), soften in 60% glacial acetic acid, dropped on slides, stained with Giemsa, and then examined under an optical microscope. For each animal, 1000 spermatogonia were examined, and the chromosomal aberrations including chromosomal number abnormality and structural alteration were recorded.

### 2.4. Subchronic Toxicity Study

The 90-day repeated-dose oral toxicity study was conducted in accordance with the test guidelines of the National Food Safety Standards of China described previously [22, 23]. The maximum tolerated dose (MTD) for oral intake of water-extract sericin was observed to be >1000 mg/kg in rats in the acute toxicity study. Therefore, 1000 mg/kg was selected as the high dose for the 90-day toxicity study.

Briefly, a total of 80 rats (60-80 g) were randomly divided into 4 groups with 10 males and 10 females in each group. The animals in the control group received deionized water at a dosing volume of 1 mL/100 g body weight (BW), while animals in the treatment groups received 1000, 500, and 250 mg/kg BW of sericin extract, respectively, for continuous 90 days. The animals were allowed free access to food and water during the exposure. Clinical signs and behavioral symptoms were recorded every day. Body weights were recorded weekly, and food consumption was recorded twice a week throughout the study. Food utilization rates were calculated as follows: Food utilization rate = body weight gain (g)/food intake (g) × 100%.

The ophthalmological examination was conducted before and at the end of the exposure. The cornea, lens, bulbar conjunctiva, and iris were observed with an ophthalmoscope.

After 90 days of the exposure, overnight fasted rats were anaesthetized with pentobarbital and sacrificed. Blood was collected immediately from the arteria abdominalis for hematological and biochemical examination, using a Sysmex XT-1800 automated hematological analyzer (Sysmex, Kobe, Japan) and an Olympus AU400 analyzer (Olympus, Tokyo, Japan), respectively. Urine samples were collected from the bladders for urinalysis using a Urit-500B urine chemistry analyzer (Urit, Guilin, China).

A complete gross necropsy was conducted on all rats. Absolute weight was measured, and relative organ weight (organ weight/body weight) was determined for the liver, spleen, kidneys, testes, ovaries, brain, heart thymus adrenal, epididymis, and uterus. The samples of organs and tissues were taken for histopathological examination, including the brain, thyroid gland, liver, spleen, pancreatic gland, heart, kidneys, adrenal gland, stomach, mesenteric lymph nodes, small intestine, jejunum, ileum, prostate, bladder, testes, and ovaries. The tissue samples were fixed in 4% neutral buffered formaldehyde, embedded in paraffin, stained with Giemsa, and then examined under a Leica DM 6000B optical microscopy (Wetzler, Germany). The number of animals with histopathological lesions was recorded, and the degree of lesions was scored into four levels: normal (0), mild (1), moderate (2), and severe (3).

### 2.5. Statistical Analysis

The data of male and female rats were analyzed separately, using SPSS v16.0 (SPSS Inc., Chicago, Illinois, USA). The homogeneity of variances of data was checked by Bartlett's test, and then, the data of the treatment groups were compared to those of the control by one-way ANOVA followed by Dunnett's test. The significance level was set at *p* ≤ 0.05.

## 3. Results

### 3.1. Characterization of the Water-Extract Sericin

The results of the amino acid analysis are shown in Table 1. The water-extract sericin contents were 1.46 g/100 mL of crude protein, 12.66 g/100 mL of free amino acids, and 1277.0 g/100 mL of hydrolyzed amino acids. Serine (26.0%), aspartic acid (16.5%), and threonine (9.8%) were the major amino acids found in the extract. The contents of tryptophan and taurine were below the detecting limits (<1.3 g/100 mL).

### 3.2. Genotoxicity Studies

The results of the bacterial reverse mutation test are shown in Table 2. Compared to the untreated group, the positive groups significantly increased the number of revertant colonies in the presence and absence of S9. At the same time, the numbers of revertant bacterial colonies in the untreated, solvent, and positive control were comparable to the historical data in our lab. On the other hand, sericin extract at doses of up to 5000 *μ*g/plate in the presence and absence of exogenous metabolic activation did not significantly affect the number of revertant bacterial colonies in all tested *S. typhimurium* strains, as compared to the untreated control. The confirmatory experiment showed similar results (data not shown).

The results of the mammalian erythrocyte micronucleus test showed that the micronucleated PCEs were significantly higher in the positive group (*p* < 0.01). In contrast, the micronucleus rate and PCEs/RBCs of mice were not affected by sericin extract, when compared to the negative control (Table 3).

For the mouse spermatogonia chromosomal aberration test, it was shown that sericin extract at all doses did not significantly affect spermatogonia chromosomal aberration in mice as compared to the negative group, while the rate of cells with the chromosomal aberration of the positive group was significantly higher than that in the negative group (*p* < 0.01, Table 4).

### 3.3. 90 d Subchronic Toxicity Study

**Figure 1:**
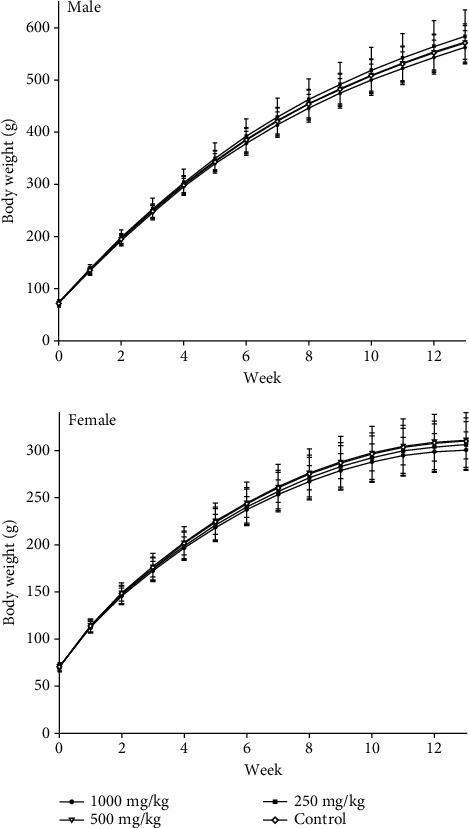
Average body weights of rats treated with water-extract sericin for 90 days (*n* = 10).

During the exposure, no treatment-related deaths or abnormal clinical changes were observed. Body weights, weight gains, food intake, and food utilization rates of the treated groups were comparable with those of the control for both sexes (*p* > 0.05; Figure 1, and Table 5). The absolute and relative weight of major organs of rats in the treated groups were similar to those of the control in both sexes (*p* > 0.05, Table 6 and Table 7). In the ophthalmological examinations, no abnormal symptoms of the eyes were observed before and after the exposure.

For blood hematology and serum biochemistry, the treatment of water-extract sericin did not induce significant changes in all parameters when compared with the control (*p* > 0.05; Table 8 and Table 9).

In urinalysis, SG, pH, urine color, and clarity of the treatment groups were comparable to those of the control (*p* > 0.05, Table 10). The positive values of WBC, PRO, BLD, Cr, Ca, and MA were observed in the treatment groups, but were considered to be spontaneous changes since the occurrences of these positive parameters were relatively low (≤30%) and comparable to those of the control.

In the gross necropsy, no apparent symptoms of pathological lesions were recorded in all animals. Therefore, histopathological examinations were conducted only in the 1000 mg/kg treatment group and the control group. In the histopathological examinations, several mild histopathological changes in the liver and kidneys of rats were observed, as listed in Table 11. Nevertheless, these histopathological changes were minor and comparable to those of the control therefore was considered to be spontaneous lesions. For other organs or tissue, no treatment-related histopathological changes were observed.

## 4. Discussion

Concerning the utilization of silk sericin, a number of studies have focused on the structural information, biological activity, and pharmacological effects [24, 25]. However, further application of sericin in food-related industries has been restricted by the limited knowledge of safety. In the present study, the toxicological safety of water-extract sericin from silkworm (*Bombyx mori*) cocoons was evaluated using different model approaches, including bacterial reverse mutation test, *in vivo* mammalian erythrocyte micronucleus test, the mouse spermatogonia chromosomal aberration test, and a 90 d repeated-dose oral toxicity study.

The amino acid composition of sericin was reported to be dependent on the extraction method used [26, 27]. In the present study, sericin was extracted by traditional boiling water extraction without chemical additives. The results of the amino acid analysis showed that the water-extract sericin contained 17 amino acids with serine accounting for 26.0% of the content. The composition and content of amino acids were consistent with the results of the previous studies using a similar extraction method and reflected the hydrophilic nature of the extract [9, 28]. Except for glycine, all amino acids exist in two mirror-like structures, which are called D- (right-handed) and L- (left-handed) enantiomers. L-amino acids are mainly involved in protein synthesis and metabolism, while D-amino acids have different and specific functions in different organisms [29]. Studies have shown that D-serine had significant nephrotoxicity in rats, inducing polyuria, diabetes, proteinuria, creatinine accumulation, and histopathological changes in the kidneys, while L-serine with the same doses did not induce nephrotoxicity ([30], Hiroshi et al. 2019). In the present study, there were no significant differences in blood urea nitrogen (BUN) and creatinine (Cr), urine protein, urine glucose, urine creatinine, kidney weight, and kidney relative weight between the treatment groups and the negative control group. In the meantime, no treatment-related histopathological changes were observed in the kidneys. These results indicated that water-extract sericin had no nephrotoxicity in rats.

Previous studies indicated that silk might be allergens leading to sensitization in humans [13, 31, 32]. It was reported that sensitization to silk was an independent predictor of childhood asthma in rural China [13]. Other studies indicated that there was a high burden of sensitization to silk allergen and occupational asthma among silk filature workers [31, 32]. Eosinophils (EO) are recognized as important cells in the process of immune response and allergic reaction. In the present study, the number of eosinophils in the rats of the treatment groups was not significantly different from that in the negative control group in the 90 d subchronic toxicity test, indicating a little sensitization effect of water-extract sericin on rats. To evaluate the genotoxicity of sericin extract, a battery of test including the bacterial reverse mutation test, the mice bone marrow micronucleus test, and the mouse spermatogonia chromosomal aberration test were conducted. The results indicated that sericin extract was nonmutagenic and nongenotoxic in both *in vitro* and *in vivo*. To further systematically evaluate the safety of water-extract sericin, a 90 d repeated-dose subchronic toxicity study was conducted. The results of the present study showed that water-extract sericin at doses of 250, 500, and 1000 mg/kg did not induce significant changes in body and organ weight, food intake, blood hematology and serum biochemistry, urine index, and histopathology of rats in both sexes. Basing on these results, the NOAEL of the water-extract sericin was determined to be 1 g/kg/day for both male and female SD rats, by oral gavage for 90 days. Using a nominal 100-fold (10-fold for differences between species and another 10-fold for differences within species) safety factor [33], the safe dose of water-extract sericin for human was determined to be 10 mg/kg/day (i.e., 0.7 g/day for a 70 kg person).

In previous studies, sericin proteins were reported to influence glucose and lipid metabolism in experimental animals [1, 28]. It was reported that 0.8% dietary sericin could reduce blood glucose levels in type 2 diabetic mice by improving the antioxidant capacity, increasing the insulin sensitivity and glycogen synthesis, and reducing the gluconeogenesis and lipid synthesis [1]. Another study reported that sericin treatment increased lipid excretion in feces in obese mice, suggesting potential antiobesity effects [28]. However, in the current study, a little effect of sericin extract on blood glucose and obesity-related parameters (e.g., body weight, cholesterol, and triglyceride) was observed. One possible explanation of this discrepancy is that the healthy rats are probably more tolerant to the effects of sericin as compared to the disease model animals. This explanation is supported by the results of a previous study showing that sericin significantly influenced the fasting blood glucose of type 2 diabetic mice while it had a little effect on the blood glucose of normal mice [1].

## 5. Conclusion

This study evaluated the safety of water-extract sericin from silkworm (*Bombyx mori*) cocoons using different model approaches, including a test of contact allergy in guinea pigs, a battery of genotoxicity study, and a 90 d subchronic toxicity study. Our results showed that water-extract sericin was nonmutagenic and nongenotoxic both *in vitro* and *in vivo*. The NOAEL of water-extract sericin was determined to be 1 g/kg/day for both male and female SD rats. These results indicated that water-extract sericin was of low toxicity in the experimental conditions of the current study and had the potential for application in food-related products.

## Figures and Tables

**Table 1:** Amino acid contents of water-extract sericin.

Amino acids	Contents (% w/w)
Aspartic acid (Asp)	16.5
Threonine (Thr)	9.8
Serine (Ser)	26.0
Glutamate (Glu)	5.0
Proline (Pro)	1.5
Glycine (Gly)	9.1
Alanine (Ala)	3.6
Cysteine (Cys)	0.3
Valine (Val)	4.2
Methionine (Met)	1.2
Isoleucine (Ile)	1.9
Leucine (Leu)	2.8
Tyrosine (Tyr)	6.3
Phenylalanine (Phe)	1.7
Lysine (Lys)	3.6
Histidine (His)	1.4
Arginine (Arg)	5.0
Total	100.0

**Table 2:** Results of the bacterial reverse mutation test for water-extract sericin.

Groups	TA97a		TA98		TA100		TA102		TA1535	
	-S9	+S9	-S9	+S9	-S9	+S9	-S9	+S9	-S9	+S9
Sericin										
5000 *μ*g/plate	117.0 ± 17.7	125.0 ± 17.5	38.3 ± 4.5	39.0 ± 4.4	157.3 ± 21.7	155.3 ± 18.6	269.0 ± 29.5	277.7 ± 22.0	24.0 ± 5.3	21.7 ± 5.5
1581 *μ*g/plate	131.3 ± 25.0	127.3 ± 22.0	39.7 ± 4.2	42.3 ± 4.2	150.7 ± 19.6	156.0 ± 16.7	280.0 ± 20.1	273.0 ± 20.1	18.3 ± 2.3	22.3 ± 5.9
500 *μ*g/plate	124.7 ± 19.7	114.7 ± 18.7	38.7 ± 7.2	41.7 ± 6.5	154.0 ± 19.7	152.0 ± 20.5	271.7 ± 22.1	266.7 ± 23.9	22.7 ± 3.2	26.7 ± 9.2
158 *μ*g/plate	123.7 ± 21.2	118.7 ± 23.7	37.7 ± 4.7	41.0 ± 4.4	152.7 ± 17.8	159.7 ± 24.0	275.7 ± 22.0	275.3 ± 21.2	22.3 ± 7.1	24.3 ± 4.2
50 *μ*g/plate	125.7 ± 16.0	120.3 ± 17.5	43.3 ± 5.0	43.0 ± 7.6	151.3 ± 21.5	156.7 ± 20.5	267.7 ± 22.9	275.7 ± 21.0	22.7 ± 7.0	25.0 ± 6.2
Untreated control	120.0 ± 13.2	130.0 ± 10.5	41.3 ± 5.5	44.3 ± 6.7	148.3 ± 22.3	155.3 ± 25.6	271.7 ± 21.2	274.7 ± 21.0	23.3 ± 6.8	23.7 ± 4.5
H_2_O control	125.0 ± 15.6	127.0 ± 19.3	41.0 ± 5.0	38.0 ± 4.6	157.3 ± 19.7	150.7 ± 19.4	271.0 ± 20.5	269.7 ± 19.8	21.7 ± 8.3	24.3 ± 5.8
DMSO control	124.3 ± 17.2	122.3 ± 17.6	36.0 ± 5.3	38.0 ± 7.0	157.7 ± 22.2	158.0 ± 21.6	279.3 ± 19.9	271.3 ± 16.3	20.0 ± 4.4	25.3 ± 8.3
Positive control										
2-AF		1785.3 ± 68.2		4886.7 ± 210.1		2886.7 ± 102.6				
DNR			2980.0 ± 111.4							
Dexon	2795.3 ± 60.5						864.7 ± 68.5			
NaN3					3056.7 ± 156.3					
1,8-DHAQ								864.3 ± 73.6		
CP									308.7 ± 13.3	139.3 ± 14.7

Values represent the mean ± standard deviation of triplicates. -S9: without metabolic activation; +S9: with metabolic activation; 2-AF: 2-aminofluorene (10 *μ*g/plate); DNR: daunorubicin (6 *μ*g/plate); Dexon: dexon (50 *μ*g/plate); NaN3: sodium azide (1.5 *μ*g/plate); 1,8-DHAQ: 1, 8-dihydroxyanthraquinone (50 *μ*g/plate); CP: cyclophosphamide (200 *μ*g/plate).

**Table 3:** Results of the mammalian erythrocyte micronucleus test for water-extract sericin.

Groups	Number of mice examined	Number of PCE examined	Number of micronucleated PCE observed	Micronucleated PCE (‰)	Number of RBC examined	Number of PCE observed	PCE/RBC
Male							
6660	5	5 × 2000	13	1.3 ± 0.4	5 × 1000	232	4.64 ± 0.92
3330	5	5 × 2000	16	1.6 ± 0.7	5 × 1000	240	4.80 ± 1.17
1665	5	5 × 2000	15	1.5 ± 0.5	5 × 1000	237	4.74 ± 0.80
Negative	5	5 × 2000	15	1.5 ± 0.5	5 × 1000	223	4.46 ± 0.72
Positive	5	5 × 2000	219	21.9 ± 4.3^∗∗^	5 × 1000	156	3.12 ± 1.12
Female							
6660	5	5 × 2000	14	1.4 ± 0.5	5 × 1000	226	4.52 ± 0.96
3330	5	5 × 2000	14	1.4 ± 0.8	5 × 1000	241	4.82 ± 1.02
1665	5	5 × 2000	15	1.5 ± 0.5	5 × 1000	224	4.48 ± 1.52
Negative	5	5 × 2000	14	1.4 ± 0.5	5 × 1000	229	4.58 ± 1.09
Positive	5	5 × 2000	213	21.3 ± 3.1^∗∗^	5 × 1000	164	3.28 ± 1.02

PCE: polychromatic erythrocyte; RBC: red blood cell. For micronucleated PCE and PCE/RBC, the data represent the mean ± standard deviation of 5 animals. ^∗∗^Significant difference compared to the control at *p* < 0.01.

**Table 4:** Results of mouse spermatogonia chromosomal aberration test for sericin extract.

Group (mg/kg)	Number of cells examined	Chromosomal number abnormality		Alteration of chromosomal structure						Number of cells with chromosomal aberration	Rate of cells with chromosomal aberration (%)
		Aneuploid	Polyploid	Gaps	Breakage	Fragment	Microchromosome	Circular	Others		
6660 (24 h)	100 × 5	0	36	28	10	26	0	1	0	101	14.6 ± 1.7
6660 (48 h)	100 × 5	0	36	22	10	24	1	0	0	93	14.2 ± 1.9
3330	100 × 5	0	33	23	10	23	0	1	0	90	13.4 ± 0.9
1665	100 × 5	0	35	32	12	12	1	1	0	93	12.2 ± 1.5
Negative control	100 × 5	0	37	20	11	24	1	1	0	92	14.4 ± 1.8
Positive control	100 × 5	0	37	47	99	164	2	7	0	356	61.8 ± 3.9^∗∗^

Rate of cells with chromosomal aberration (%) = (number of cells with chromosomal aberration − number of cells with chromosomal gaps)/number of cells examined × 100%.

**Table 5:** Body weight gain, food intake, and food utilization rate of rats treated with sericin extract for 90 days.

Sex	Dose (mg/kg)	Total body weight gain (g)	Total food intake (g)	Average food utilization rate (%)
Male	1000	489.6 ± 32.1	2604.2 ± 145.3	18.8 ± 1.0
	500	498.1 ± 31.6	2673.0 ± 168.4	18.7 ± 1.0
	250	511.1 ± 46.9	2669.3 ± 205.8	19.2 ± 0.6
	Control	497.5 ± 36.4	2701.7 ± 143.5	18.4 ± 0.8

Female	1000	230.7 ± 16.8	1798.0 ± 104.5	12.9 ± 0.9
	500	240.5 ± 17.6	1813.2 ± 113.5	13.3 ± 0.6
	250	236.2 ± 28.2	1823.2 ± 164.5	13.0 ± 0.7
	Control	238.9 ± 29.2	1852.4 ± 122.5	12.9 ± 1.1

Note: The values represent the mean ± standard deviation of 10 rats. Food utilization rate (%) = (total body weight gain/total food consumption) × 100%. The values of the treatment groups did not differ statistically from the control according to one-way ANOVA at *p* < 0.05.

**Table 6:** Absolute organ weights of rats treated with sericin extract for 90 days.

Dose (mg/kg)	Liver	Kidneys	Spleen	Testes/ovaries	Brain	Heart	Thymus	Adrenal	Epididymis/uterus
Male									
1000	13.83 ± 1.08	3.532 ± 0.229	0.939 ± 0.050	3.715 ± 0.309	2.168 ± 0.066	1.561 ± 0.104	0.590 ± 0.078	0.085 ± 0.016	1.348 ± 0.112
500	13.54 ± 0.99	3.479 ± 0.266	0.946 ± 0.074	3.673 ± 0.253	2.164 ± 0.078	1.627 ± 0.124	0.540 ± 0.067	0.083 ± 0.013	1.431 ± 0.120
250	14.05 ± 1.98	3.599 ± 0.504	0.961 ± 0.098	3.728 ± 0.401	2.099 ± 0.075	1.663 ± 0.200	0.591 ± 0.074	0.090 ± 0.026	1.389 ± 0.185
Control	13.92 ± 1.36	3.573 ± 0.243	0.925 ± 0.094	3.742 ± 0.155	2.085 ± 0.098	1.639 ± 0.129	0.541 ± 0.068	0.084 ± 0.016	1.446 ± 0.175

Female									
1000	7.74 ± 0.89	1.873 ± 0.135	0.555 ± 0.075	0.158 ± 0.023	1.899 ± 0.046	1.015 ± 0.084	0.433 ± 0.051	0.075 ± 0.015	0.594 ± 0.064
500	7.56 ± 0.45	1.909 ± 0.121	0.597 ± 0.052	0.159 ± 0.015	1.967 ± 0.079	1.005 ± 0.101	0.477 ± 0.057	0.072 ± 0.010	0.657 ± 0.077
250	7.93 ± 1.16	1.883 ± 0.173	0.559 ± 0.046	0.159 ± 0.029	1.896 ± 0.094	1.006 ± 0.064	0.458 ± 0.053	0.076 ± 0.013	0.609 ± 0.065
Control	7.96 ± 1.08	1.965 ± 0.350	0.571 ± 0.055	0.155 ± 0.018	1.890 ± 0.070	0.999 ± 0.148	0.455 ± 0.085	0.073 ± 0.012	0.624 ± 0.091

Note: The values represent the mean ± standard deviation of 10 rats. The values of the treatment groups did not differ statistically from the control according to one-way ANOVA at *p* < 0.05.

**Table 7:** Relative organ weights of rats treated with sericin extract for 90 days.

Dose (mg/kg)	Liver	Kidneys	Spleen	Testes/ovaries	Brain	Heart	Thymus	Adrenal	Epididymis/uterus
Male									
1000	2.562 ± 0.173	0.655 ± 0.051	0.174 ± 0.011	0.689 ± 0.057	0.402 ± 0.019	0.289 ± 0.018	0.109 ± 0.010	0.016 ± 0.002	0.250 ± 0.026
500	2.475 ± 0.118	0.636 ± 0.039	0.173 ± 0.016	0.673 ± 0.048	0.397 ± 0.023	0.298 ± 0.021	0.098 ± 0.009	0.015 ± 0.002	0.263 ± 0.030
250	2.488 ± 0.149	0.638 ± 0.052	0.171 ± 0.009	0.663 ± 0.045	0.375 ± 0.025	0.296 ± 0.026	0.105 ± 0.010	0.016 ± 0.004	0.247 ± 0.026
Control	2.531 ± 0.133	0.652 ± 0.051	0.169 ± 0.016	0.684 ± 0.058	0.380 ± 0.018	0.299 ± 0.021	0.098 ± 0.010	0.015 ± 0.003	0.264 ± 0.036

Female									
1000	2.696 ± 0.164	0.654 ± 0.041	0.193 ± 0.021	0.055 ± 0.009	0.665 ± 0.042	0.355 ± 0.031	0.151 ± 0.018	0.026 ± 0.004	0.208 ± 0.025
500	2.534 ± 0.157	0.640 ± 0.046	0.201 ± 0.021	0.054 ± 0.008	0.661 ± 0.053	0.337 ± 0.033	0.160 ± 0.016	0.024 ± 0.004	0.221 ± 0.030
250	2.688 ± 0.189	0.641 ± 0.040	0.191 ± 0.019	0.054 ± 0.007	0.648 ± 0.054	0.344 ± 0.03	0.156 ± 0.019	0.026 ± 0.004	0.209 ± 0.034
Control	2.694 ± 0.121	0.663 ± 0.056	0.195 ± 0.018	0.053 ± 0.008	0.646 ± 0.051	0.340 ± 0.038	0.154 ± 0.022	0.025 ± 0.003	0.213 ± 0.035

Note: The values represent the mean ± standard deviation of 10 rats. Relative organ weight (%) = (absolute organ weight/fasting body weight) × 100%. The values of the treatment groups did not differ statistically from the control according to one-way ANOVA at *p* < 0.05.

**(a):** 

Sex	Dose (mg/kg)	HGB (g/L)	RBC (10^12^/L)	PLT (10^9^/L)	HCT (%)	PT (s)	APTT (S)
Male	1000	156.0 ± 6.5	8.54 ± 0.37	982.4 ± 119.9	44.4 ± 1.6	9.3 ± 0.6	15.6 ± 1.7
	500	155.2 ± 3.8	8.34 ± 0.26	946.6 ± 138.1	44.0 ± 2.1	9.5 ± 0.5	15.6 ± 1.1
	250	154.4 ± 6.4	8.36 ± 0.32	958.6 ± 132.2	43.2 ± 2.0	9.3 ± 0.5	16.3 ± 1.9
	Control	155.8 ± 5.5	8.49 ± 0.31	966.6 ± 149.0	44.1 ± 2.5	9.2 ± 0.4	16.1 ± 1.2

Female	1000	154.8 ± 8.0	8.18 ± 0.35	946.8 ± 126.4	44.5 ± 2.1	9.2 ± 0.6	15.8 ± 1.7
	500	149.6 ± 7.8	7.88 ± 0.36	998.8 ± 110.4	42.5 ± 2.1	9.2 ± 0.5	16.0 ± 1.6
	250	155.4 ± 6.0	8.33 ± 0.30	953.4 ± 157.3	44.6 ± 2.1	9.4 ± 0.6	15.9 ± 1.0
	Control	151.6 ± 9.3	8.01 ± 0.43	985.4 ± 169.9	43.7 ± 2.0	9.3 ± 0.4	15.7 ± 1.2

**(b):** 

Sex	Dose (mg/kg)	WBC (10^9^/L)	LYM (%)	NEUT (%)	MONO (%)	EO (%)	BASO (%)
Male	1000	7.81 ± 1.09	74.6 ± 5.3	18.7 ± 4.8	4.90 ± 0.82	1.79 ± 0.47	0.02 ± 0.06
	500	8.19 ± 1.37	73.5 ± 4.8	19.9 ± 4.2	5.05 ± 0.83	1.55 ± 0.60	0.04 ± 0.08
	250	8.72 ± 1.74	74.3 ± 5.3	19.2 ± 5.6	4.89 ± 0.63	1.61 ± 0.47	0.02 ± 0.06
	Control	8.49 ± 1.38	73.8 ± 4.4	19.2 ± 4.6	5.14 ± 0.62	1.89 ± 0.45	0.04 ± 0.08

Female	1000	7.74 ± 1.19	75.3 ± 3.4	18.2 ± 3.9	4.40 ± 0.55	2.05 ± 0.52	0.02 ± 0.06
	500	8.19 ± 0.86	76.1 ± 5.2	17.1 ± 5.0	5.02 ± 0.78	1.77 ± 0.80	0.02 ± 0.06
	250	8.06 ± 1.35	74.4 ± 5.2	18.5 ± 5.1	4.97 ± 0.62	2.11 ± 0.54	0.03 ± 0.09
	Control	7.76 ± 1.20	75.5 ± 4.6	17.8 ± 4.5	4.82 ± 0.52	1.82 ± 0.60	0.04 ± 0.08

Note: The values represent the mean ± standard deviation of 10 rats. HGB: hemoglobin concentration; RBC: red blood cell count; PLT: platelet count; HCT: hematocrit; PT: prothrombin time; APTT: activated partial thromboplastin time; WBC: white blood cell count; LYM: percent of lymphocytes; NEUT: percent of neutrophils; MONO: percent of monocytes; EO: percent of eosinophils; BASO: percent of basophils. The values of the treatment groups did not differ statistically from the control according to one-way ANOVA at *p* < 0.05.

**(a):** 

Dose (mg/kg)	AST (U/L)	ALT (U/L)	BUN (mmol/L)	CR (*μ*mol/L)	TC (mmol/L)	TG (mmol/L)	TP (g/L)
Male							
1000	100.4 ± 9.9	42.5 ± 6.0	7.10 ± 0.64	34.6 ± 4.7	1.84 ± 0.3	0.82 ± 0.17	68.0 ± 2.5
500	105.8 ± 13.0	50.4 ± 4.8	7.09 ± 0.85	36.3 ± 4.6	1.76 ± 0.2	0.93 ± 0.19	67.7 ± 3.3
250	99.8 ± 12.0	45.4 ± 6.8	6.38 ± 0.68	33.7 ± 4.8	1.80 ± 0.3	0.85 ± 0.20	67.8 ± 3.6
Control	96.3 ± 10.4	46.3 ± 5.2	6.54 ± 0.57	34.9 ± 5.1	1.94 ± 0.2	0.98 ± 0.19	66.7 ± 2.5

Female							
1000	91.3 ± 6.0	41.8 ± 3.5	6.93 ± 0.77	39.0 ± 5.9	1.98 ± 0.3	0.88 ± 0.14	74.6 ± 4.4
500	88.8 ± 7.4	43.3 ± 3.3	6.79 ± 0.66	40.0 ± 4.4	2.07 ± 0.4	0.91 ± 0.20	72.7 ± 4.2
250	95.2 ± 8.6	42.1 ± 6.0	6.94 ± 0.54	37.4 ± 5.8	2.09 ± 0.4	0.91 ± 0.27	75.9 ± 4.0
Control	90.1 ± 8.9	40.3 ± 5.4	6.66 ± 0.80	38.6 ± 5.2	2.22 ± 0.3	0.94 ± 0.25	73.2 ± 4.1

**(b):** 

Dose (mg/kg)	ALB (g/L)	GLU (mmol/L)	GGT (U/L)	ALP (U/L)	Cl (mmol/L)	K (mmol/L)	Na (mmol/L)
Male							
1000	35.4 ± 1.7	5.72 ± 0.60	0.84 ± 0.32	127.6 ± 15	109.3 ± 0.9	6.42 ± 0.39	181.6 ± 3.1
500	35.4 ± 2.2	5.58 ± 0.44	0.87 ± 0.29	133.4 ± 11	108.7 ± 1.5	6.54 ± 0.29	181.7 ± 3.6
250	34.8 ± 1.8	5.92 ± 0.53	0.85 ± 0.34	126.0 ± 15	108.2 ± 1.8	6.26 ± 0.31	180.2 ± 4.1
Control	35.0 ± 1.4	5.97 ± 0.59	0.78 ± 0.32	132.7 ± 14	107.8 ± 1.5	6.20 ± 0.31	179.6 ± 3.1

Female							
1000	38.5 ± 2.8	5.57 ± 0.40	0.73 ± 0.26	57.5 ± 7.9	107.7 ± 2.3	6.06 ± 0.25	181.5 ± 4.2
500	36.8 ± 2.8	6.09 ± 0.43	0.84 ± 0.19	63.7 ± 9.3	109.3 ± 2.0	6.32 ± 0.42	183.4 ± 4.4
250	39.6 ± 3.1	5.99 ± 0.31	0.77 ± 0.14	60.0 ± 8.4	106.6 ± 3.0	6.15 ± 0.30	181.2 ± 2.4
Control	37.7 ± 3.0	5.86 ± 0.56	0.78 ± 0.25	61.5 ± 8.9	107.9 ± 2.2	6.14 ± 0.31	183.0 ± 2.9

Note: The values represent the mean ± standard deviation of 10 rats. AST: aspartate transaminase; ALT: alanine transaminase; BUN: blood urea nitrogen; CR: creatinine; TC: total cholesterol; TG: triglyceride; TP: total protein; ALB: albumin; GLU: glucose; GGT: glutamyltransferase; ALP: alkaline phosphatase; Cl: chlorine; K: potassium; Na: Natrium. The values of the treatment groups did not differ statistically from the control according to one-way ANOVA at *p* < 0.05.

**Table 10:** Urinalysis of rats treated with sericin extract for 90 days.

Dose (mg/kg)	Number of abnormal color/clarity	SG	pH	Number of positive										
				WBC	KET	NIT	URO	BIL	PRO	GLU	BLD	Cr	Ca	MA
Male														
1000	0	1.014 ± 0.003	7.7 ± 0.3	0	0	0	0	0	3	0	2	0	2	2
500	0	1.016 ± 0.003	7.6 ± 0.5	2	0	0	0	0	3	0	2	1	2	3
250	0	1.014 ± 0.003	7.5 ± 0.4	1	0	0	0	0	2	0	2	0	1	2
Control	0	1.013 ± 0.003	7.7 ± 0.3	2	0	0	0	0	3	0	2	1	2	2
Female														
1000	0	1.015 ± 0.004	7.6 ± 0.4	0	0	0	0	0	1	0	1	0	2	1
500	0	1.015 ± 0.004	7.4 ± 0.5	1	0	0	0	0	2	0	1	0	1	1
250	0	1.014 ± 0.004	7.6 ± 0.4	0	0	0	0	0	0	0	2	0	1	1
Control	0	1.014 ± 0.003	7.5 ± 0.3	1	0	0	0	0	2	0	2	0	2	1

Note: The values of SG and pH represent the mean ± standard deviation of 10 rats. SG: specific gravity; WBC: white blood cell; KET: ketone body; NIT: nitrite; URO: urobilinogen; BIL: bilirubin; PRO: protein; GLU: glucose; BLD: occult blood; Cr: creatinine; Ca: calcium; MA: microalbumin. The values of the treatment groups did not differ statistically from the control according to one-way ANOVA at *p* < 0.05.

**Table 11:** Histopathology examination of rats treated with sericin extract for 90 days.

Organs	Histopathological changes	Male		Female	
		1000 mg/kg	Control	1000 mg/kg	Control
Liver	Inflammatory cell infiltration in portal duct areas	2	1	1	2
	Mild spotty necrosis of hepatocytes	1	1	1	1
	Mild fatty degeneration of hepatocytes	1	2	2	2
Kidneys	Cell infiltration in renal cortex	1	2	2	1

Note: The values represent the number of rats with histopathological changes in 10 rats of each group.

## Data Availability

The data that support the findings of this study are available from the corresponding author upon request.
